# Does happiness matter to health system efficiency? A performance analysis

**DOI:** 10.1186/s13561-018-0214-6

**Published:** 2018-12-21

**Authors:** Kok Fong See, Siew Hwa Yen

**Affiliations:** 1 Economics Programme, School of Distance Education, Universiti Sains Malaysia, Pulau Pinang, Malaysia; 2 Centre for the Advancement of the Humanities and Social Sciences, National Taiwan University, Taipei City, Taiwan

**Keywords:** Global health system, Happiness, International benchmarking, Technical efficiency, Data envelopment analysis

## Abstract

**Background:**

The main objective of this study is to assess the performance of the global health system, emphasising the contribution of people’s happiness to health system efficiency across countries. Technical efficiency (TE) scores are estimated using the output-oriented variable returns to scale (VRS) data envelopment analysis (DEA) model based on the input measures: health expenditure, labour, hospital beds and education, and the output measures: healthy life expectancy and inverse mortality index. The efficiency scores are regressed against three explanatory variables: happiness index, population density, and healthcare share of gross domestic product (GDP). The analysis involved 121 selected countries using double bootstrap DEA as proposed by Simar, L., Wilson, P.W J Econ 136:1‑34, 2007.

**Results:**

The bootstrap truncated regression indicates that happiness is one of the factors that contributes significantly to health system efficiency. The study also revealed that the selected health systems perform well, on average, in terms of population density and healthcare share of GDP.

**Conclusions:**

In addition to improving the economic standard of living, policy-makers should also consider ways to increase the happiness and well-being of society. Policies focusing on well-being and happiness can lead to improved well-being and improved health outcomes, which may ultimately reduce the healthcare burden and enhance healthcare performance.

**Electronic supplementary material:**

The online version of this article (10.1186/s13561-018-0214-6) contains supplementary material, which is available to authorized users.

## Introduction

A considerable amount of research has recognised health as one of the most important correlates of happiness and well-being. Therefore, whether it has implications for health policies is a pertinent question. Can policies aimed at increasing happiness improve health and ultimately reduce the healthcare burden? Many studies have consistently revealed a strong relationship between health and happiness. Such a relationship is found to be more statistically robust than that between happiness and income. Good health is linked to higher happiness levels, and causality seems to run in both directions.

Literature reviews and meta-analyses on happiness and health have generally concluded that happiness or subjective well-being can be beneficial to health and longevity [1, 2]. Conversely, good health is often recognised as the prerequisite to achieve happiness and well-being [3, 4]. In fact, mental health is found to be more important in determining well-being than income [5]. Negative emotions aggravate cardiovascular activity, whereas positive emotions help accelerate physiological recovery to desirable levels [6]. People with negative emotional style tend to have poorer immune system and increased risk of illness compared with those with positive emotional style [7].

High levels of subjective well-being or happiness can increase life expectancy by four to ten years compared with the life expectancy associated with low levels of subjective well-being. This outcome was obtained even after controlling for initial health conditions such as depression, anxiety, coronary heart disease and cancer [8]. Chida and Steptoe [9] showed a significant reduction of all-cause mortality among people with higher levels of well-being. Conversely, low levels of happiness might cause some people to engage in activities that are known to affect their health adversely, such as smoking, drinking, being dependent on drugs or being physically inactive [10, 11].

Bhutan, as the first country in the world to pursue happiness as a state policy, recognises health as one of the most important means to achieving gross national happiness [12]. However, so far, there is no study that specifically addresses the association of people’s happiness with the nation’s health system performance. Happiness index is a comprehensive indicator that reflects the quality of the society and should not be ignored.^1^ As proven in many studies good health promotes higher levels of happiness and happiness promotes better health. Improved well-being and improved health outcomes may ultimately improved well-being and improved health outcomes may ultimately lead to reduction in the healthcare burden and improved healthcare performance.

The main objective of this study is to assess the performance of global health system using double bootstrap data envelopment analysis (DEA) approach. This study emphasises the contribution of people’s happiness and well-being on health system performance across 121 countries. Health system technical efficiency scores will be generated for each countries using DEA model based on relevant input and output measures. The efficiency scores will then be regressed against a set of explanatory variables that includes happiness index, population density and healthcare share of gross domestic product (GDP). In general, meta-analyses of the scientific literature have concluded that subjective well-being can be beneficial to health and longevity [1]. Better health outcomes could lessen healthcare costs and eventually improved the performance. Therefore, happiness index could be one of the environmental factors which will be relevant in explaining the variation in global health system performance.

Several empirical efficiency studies related to the health system are discussed in Section 2. Section 3 briefly describes the double bootstrap DEA model and the variable selected in the study. The research findings are highlighted in Section 4. A brief conclusion is given in Section 5.

## Insights from health system efficiency studies

Most of the empirical studies on health system efficiency and performance involved the analysis of data from individual countries. Most of these studies were carried out in developed countries, for instance, some recent studies in Canada by Allin et al. [13], Spain by Carrillo and Jorge [14] and Greece by Mitropoulos et al. [15]. However, such studies may not be appropriate when there is an insufficient number of similar health service providers available within a country, especially for developing countries. To assess overall health system performance and make comparisons across countries, a global health system benchmarking study may be essential. Undoubtedly, the potential benefits of international benchmarking, such as improving the validity of analysis, largely outweigh the costs.

A summary of previous efficiency studies on health system performance is provided in Table A1 (Please refer to Additional file 1: Appendix A). As shown in Table A1, two main approaches used in the analysis are DEA and stochastic frontier analysis (SFA), which involved numerous combinations of input variables. Most health system performance studies focused on Organisation for Economic Co-operation and Development (OECD) countries, and only a few covered global perspectives, partly because more comprehensive healthcare data are reported in the OECD countries and made accessible to the public. Performance studies of the health system among the OECD countries include the works of de Cos and Moral-Benito [16], Hadad et al. [17], Wranik [18], Afonso and Aubyn [19], Spinks and Hollingsworth [20], Afonso and Aubyn [21], Bhat [22] Osterkamp [23], Retzlaff-Roberts et al. [24] and Puig-Junoy[25].

Hadad et al. [17], for instance, employed an output-oriented DEA approach to examine the health system efficiency of 31 OECD countries in 2007. The input variables were defined as fruit and vegetable consumption, health spending, hospital beds, income and physicians, whereas infant mortality and life expectancy were defined as the output measures. Fat intake, public health expenditure, unemployment, income inequality, environmental health score, organisational arrangements, public health objectives, gatekeeping, and performance-related payment incentives were included in the second-stage analysis. The outcomes of that study met prior expectations and provided further support for the hypothesis that all conditional variables are significant for health system efficiency.

A similar study was performed by Afonso and Aubyn [19] to examine health system efficiency and identify factors that affect performance. Their study used panel data from 2001 to 2002 to benchmark the performance of the health systems in 21 OECD countries. The inputs for the model were hospital beds, MRI, physicians and nurses, while the outputs were infant mortality, life expectancy and potential years of life lost. The environmental factors adopted by the authors included income, education, obesity, and tobacco consumption. By using the algorithm #1 double bootstrap DEA approach, the authors found that all selected explanatory variables were significantly correlated with health system performance. Notably, although algorithm #1 is a valid procedure, some inconsistencies in the analysis could arise if strong parametric assumptions were made in one step of the analysis but avoided in others.

Over the last decade, several efficiency studies have also been conducted to examine the global health system, including Sinimole [26], Ogloblin [27], Greene [28, 29], Kumbhakar [30], Grosskopf et al. [31], and Hollingsworth and Wildman [32], that measured the performance of the global health system based on World Health Organisation (WHO) data. For instance, Ogloblin [27] examined the effects of income, income inequality, and health expenditure on the health system efficiency of 78 countries based on pooled data in 2000, 2003 and 2007. SFA was used in the analysis, with the output measure defined as health-adjusted life expectancy. The input measures involved in their analysis were health expenditure, education, tobacco consumption and alcohol consumption. The results indicated that income as well as public and private health expenditures were positively related to health system efficiency, whereas income inequality seemed to have an adverse effect.

A similar study from the global perspective was carried out by Kumbhakar [30] based on data from 180 nations between 1993 and 1997. That study used an SFA approach to benchmark the performance of those 180 health systems. Health expenditure and education levels were used as the input measures and disability-adjusted life expectancy as the output measure in that study. Several explanatory variables, including income inequality, democratisation and freedom index, government effectiveness, tropical location, population density, income, and OECD membership, were hypothesised to affect health system performance. The findings indicated that income, income inequality, government effectiveness and population density were significantly correlated with health system performance. Overall, as indicated in Table A1 (Additional file 1: Appendix A), health expenditure and education levels were commonly chosen as the input measures, while life expectancy and infant mortality as the output measures.

## Methods

The task of measuring healthcare performance can be carried out by using these two main techniques, which are, SFA and DEA. The SFA model applies an econometric method that involves a functional form to estimate efficiency. On the other hand, DEA which is a frontier method uses linear programming technique to determine relative efficiency of each decision making unit (DMU) within a set of homogeneous DMUs. A standard DEA model measures efficiency as the ratio of the sum of its weighted outputs to the sum of its weighted inputs. It is particularly useful in handling efficiency analysis for public utilities and public organisations such as healthcare facilities which are not profit oriented and employ a multiple input-multiple output production function. Based on previous published studies, DEA model has been proven to be effective in identifying the best practice in a sample by constructing a piece-wise linear production frontier.

### Standard DEA model

DEA model can be specified using either an input or output oriented approach. This study adopts the output-oriented variable returns to scale (VRS) DEA model to measure technical efficiency (TE) scores. Assuming that data on K inputs and M outputs are available for N countries' health systems involved in the study. The notation X represents the K × N matrix of inputs, consisting of K inputs from N countries, while Y represents the M × N matrix of outputs, consisting of M outputs from N countries. The output-oriented VRS DEA model is commonly presented in the envelopment form as below:

The output-oriented VRS model

1 $$ {\displaystyle \begin{array}{l}{\operatorname{Max}}_{\uptheta, \uplambda}\ \uptheta, \\ {}\mathrm{Subject}\ \mathrm{to}:\\ {}\hbox{-} \uptheta \mathrm{yi}+\mathrm{Y}\uplambda \ge 0,\\ {}\mathrm{xi}\hbox{-} \mathrm{X}\uplambda \ge 0,\\ {}\mathrm{N}{1}^{'}\uplambda =1\\ {}\uplambda \ge 0\end{array}} $$

θ and λ denote a scalar and an N × 1 vector of weights respectively. The value of θ ranges from one to infinity, and θ − 1 is the proportional output expansions, which can be attained by the i^th^ country, given the input level. See Ozcan [33] for more details on the DEA methodology in healthcare studies.

### Double bootstrap DEA approach

In many DEA literature, Tobit regression is being employed to account for the effects of explanatory variables on the technical efficiency. In the first stage, the technical efficiency scores are estimated through DEA model and the scores will then be regressed against a set of explanatory variables as follows:

2 $$ {\uptheta}_{\mathrm{m}}=\mathrm{a}+{\upbeta \mathrm{z}}_{\mathrm{m}}+{\upvarepsilon}_{\mathrm{m}} $$

where ${\uptheta}_{\mathrm{m}}$ is the technical efficiency score, z_m_ is the vector of variables that influences the technical efficiency of health system, β is a vector of the parameters to be estimated, and ε_m_ is an error term where the value of ε_m_ is greater than or equal to the sum of 1 − a − βz_m_. The second stage of analysis offers useful information to enhance the operating environment for health system and design better incentive schemes for internal improvement. However, Simar and Wilson [34] found that the DEA results are greatly dependent on each other and therefore, using them in the second stage regression is inappropriate as it would violate the basic assumption of regression models.

Simar and Wilson [34] demonstrated that the double bootstrap DEA approach can be a better choice.^2^ Thus, in this study, the double bootstrap DEA approach is employed to analyse the determinants of global health system performance. Algorithm #2 as suggested by Simar and Wilson [34] used in the study is as follows:

- Step 1: Compute the technical efficiency scores $\left({\widehat{\uptheta}}_{\mathrm{m}}\right)$ using DEA model.
- Step 2: Regress the technical efficiency scores on z_m_ using truncated regression to obtain the parameter estimates $\left(\widehat{\upbeta},{\widehat{\upsigma}}_{\upvarepsilon}\right)$ by the maximum likelihood.
- Step 3: Repeat the following steps: 3.1–3.4 by B_1_ times to obtain a set of bootstrap estimates:
  3.1 Draw ${\upvarepsilon}_{\mathrm{m}}^{\ast }$ from a normal distribution, $\mathrm{N}\left(0,{\widehat{\upsigma}}_{\upvarepsilon}\right)$, with a left truncation at $\left(1-\widehat{\upbeta}{\mathrm{z}}_{\mathrm{m}}\right)$ for all m = 1,…,M;
  3.2 Calculate ${\uptheta}_{\mathrm{m}}^{\ast }=\widehat{\upbeta}{\mathrm{z}}_{\mathrm{m}}+{\upvarepsilon}_{\mathrm{m}}^{\ast }$ for all m = 1,…,M;
  3.3 Generate pseudo data (${\mathrm{x}}_{\mathrm{m}}^{\ast }={\mathrm{x}}_{\mathrm{m}}$,${\mathrm{y}}_{\mathrm{m}}^{\ast }={\mathrm{y}}_{\mathrm{m}}{\widehat{\uptheta}}_{\mathrm{m}}/{\uptheta}_{\mathrm{m}}^{\ast }$) to form the reference bootstrap for all m = 1,…,M; and
  3.4 Calculate the bootstrap estimate technical efficiency scores $\left({\widehat{\uptheta}}_{\mathrm{m}}^{\ast}\right)$ using pseudo data where X and Y are replaced by ${\mathrm{X}}^{\ast }=\left[{\mathrm{x}}_1^{\ast}\dots {\mathrm{x}}_{\mathrm{m}}^{\ast}\right]$ and ${\mathrm{Y}}^{\ast }=\left[{\mathrm{y}}_1^{\ast}\dots {\mathrm{y}}_{\mathrm{m}}^{\ast}\right]$, respectively.
- Step 4: Calculate the bias-corrected estimate, $\widehat{\widehat{\uptheta}}$ by computing the difference between the original estimates and the bootstrap estimator of bias.
- Step 5: Regress the bias-corrected estimator on z_m_ using truncated regression to obtain the parameter estimates $\left(\widehat{\widehat{\upbeta}},\widehat{\widehat{\upsigma}}\right)$ by maximum likelihood.
- Step 6: Repeat the following steps: 6.1–6.3 by B_2_ times to obtain a set of bootstrap estimates:

6.1 Draw ${\upvarepsilon}_{\mathrm{m}}^{\ast }$ from a normal distribution, N$\left(0,{\widehat{\widehat{\upsigma}}}_{\upvarepsilon}\right)$, with a left truncation at $\left(1-\widehat{\widehat{\upbeta}}{\mathrm{z}}_{\mathrm{m}}\right)$ for all m = 1,…,M;
6.2 Calculate ${\uptheta}_{\mathrm{m}}^{\ast \ast }=\widehat{\widehat{\upbeta}}{\mathrm{z}}_{\mathrm{m}}+{\upvarepsilon}_{\mathrm{m}}^{\ast \ast }$ for all m = 1,…,M; and
6.3 Regress ${\uptheta}_{\mathrm{m}}^{\ast \ast }$ on z_m_ using truncated regression to obtain the parameter estimates $\left({\widehat{\widehat{\upbeta}}}^{\ast },{\widehat{\widehat{\upsigma}}}^{\ast}\right)$by maximum likelihood.

- Step 7: Construct confidence intervals and standard errors from the bootstrap results.

### Selections of input and output measures

Most health system efficiency studies require time-consuming data collection. Obtaining reliable and sufficient data has always been a challenge. A more comprehensive and effective study of efficiency should include data over a certain time period. The input and output measures used in previous studies are summarised in Table A1 (Additional file 1). Health expenditures, physicians and hospital beds are commonly selected as inputs, while life expectancy and infant mortality are chosen as outputs in most previous studies of health system efficiency (e.g. [17, 24, 35, 36]). To adhere to DEA requirements, the minimum observations should be greater than or equal to three times the total of input and output variables. Considering the literature survey, this study uses the following input measures.

#### Health expenditures

The total health expenditures from government, private and other resources are important to deliver healthcare services to the nation. The variable is measured in million dollar-based PPP in 2011.

#### Labour

Physicians as well as nursing and midwifery personnel are the common health system staff considered in previous studies. Information related to other allied health professions for each country is usually not publicly available. To compare health system performance, the total numbers of physicians as well as nursing and midwifery personnel are used in this study.

#### Hospital beds

The total number of hospital beds is used as proxy for hospital size and hospital capital measures. To reflect the capital measure used in the health system, this analysis uses the number of hospital beds per 1000 people.

#### Education

The average school age is used as a proxy for the level of education for each country. It is strongly believed that the level of education is positively related to the average life expectancy. Therefore, education input is used as one of the inputs in the health system.

Two outputs, which are, healthy life expectancy and inverse mortality rate, are used in this study.

#### Healthy life expectancy

This measure, also known as disability-free life expectancy, is measured by the number of remaining years that a person of a certain age is still expected to live without disability. Healthy life expectancy is a better measure that introduces quality of life, as compared to standard life expectancy, which is exclusively centred on the length of life.

#### Inverse mortality index

Mortality per 1000 live births is a measure of the number of deaths in a particular population. Three types of mortality rate are published by the World Health Organisation. To meet the rule of thumb, the infant mortality rate is chosen in the study because this mortality rate is relatively higher in health systems with poor performance. Since this measure is used as one of the outputs in the health system, the reciprocal of the mortality rate is a preferable measurement. A higher inverse mortality rate is better for the health system of the country.

In addition, three explanatory variables were identified as possible determinants associated with variations in health system efficiency.

#### Happiness level

The happiness index has been published in the World Happiness Report since 2012. It is commonly used to represent the collective happiness of the people in a nation. Several important components, including caring, freedom, generosity, honesty, health, income and good governance, are adopted to construct the happiness index. To avoid the double counting effect in the second stage of the double bootstrap DEA framework, several explanatory variables that are highly correlated with happiness (e.g., income, income inequality) are excluded from the analysis. Countries with happier people are expected to have more efficient health systems.

#### Population density

This variable is measured as the ratio of the total population served to the total land area. Several empirical studies, including those by Greene [28] and Kumbhakar [30], have confirmed that population density has a significant effect on health system performance. A low population density implies a smaller population served over a large area. Hence, it is not surprising that a health system with areas with a higher population density may appear to be more efficient.

#### Healthcare share of GDP

Total health expenditures as a share of GDP vary markedly across developed and developing countries. The relationship between expenditures on healthcare and health outcomes may be unclear, especially for some developed countries, because some countries with a higher healthcare share of GDP do not always obtain better health outcomes, such as longer life expectancy.

## Results and discussion

Table 1 presents the descriptive statistics for the variables used in the study. Global health system performance is estimated based on a five inputs and two outputs model. The total number of countries involved in this study is 121 (representing nearly 75% of total global health expenditures per capita in 2014), and these countries were chosen based on the availability of relevant data required for the analysis. As shown, in terms of labour, the average number of physicians is 64,382 persons, and the average number of nursing and midwifery personnel is 139,525 persons. The number of hospital beds is used as a proxy for capital in the study. The average number of hospital beds is recorded as 3.08 per 1000 people, with a standard deviation of 2.44 per 1000 people. The average years of schooling and health expenditures are 8.88 years and 44,609.04 million dollar-based PPPs, respectively. For the output measures, the average healthy life expectancy is 64.48 years, while the average inverse mortality rate is 141.72.

**Table 1:** Descriptive statistics: Global health care statistics

Variable	Unit measurement	Mean	Std. Dev.	Min	Max
Physicians	Headcount	64,382.71	208,492.50	51.00	2,020,154.00
Nursing and midwifery personnel	Headcount	139,525.90	373,821.60	570.00	2,587,719.00
Education	Years	8.88	3.04	1.40	13.40
Health expenditures	million $PPPs	44,609.04	117,834.10	112.88	997,281.40
Hospital beds	Per 1000 people	3.08	2.44	0.10	13.70
Healthy life expectancy	Years	64.48	6.57	45.90	74.90
Inverse mortality rate	Value	141.72	139.20	10.70	588.24

### Original DEA scores, bias and bias-corrected efficiency scores

The efficiency scores for the selected global health systems in this study were calculated using the R package. The results of the DEA original efficiency scores, bias and bias-corrected efficiency scores for each country’s health system are presented in Table A2 (Additional file 2: Appendix B). The output-oriented VRS DEA model is used to calculate efficiency scores for the selected global health systems. The efficiency scores range from one to infinity, where one implies that the country's health system is efficient and lies on the production frontier. The average bias-corrected technical score is 1.036 on average, with biases of 1.52%, ranging from 0.30% to 3.54%. From Table A2 in Additional file 2, we can observe that the original DEA scores do not fall within the 95% confident intervals.

**Fig. 1:**
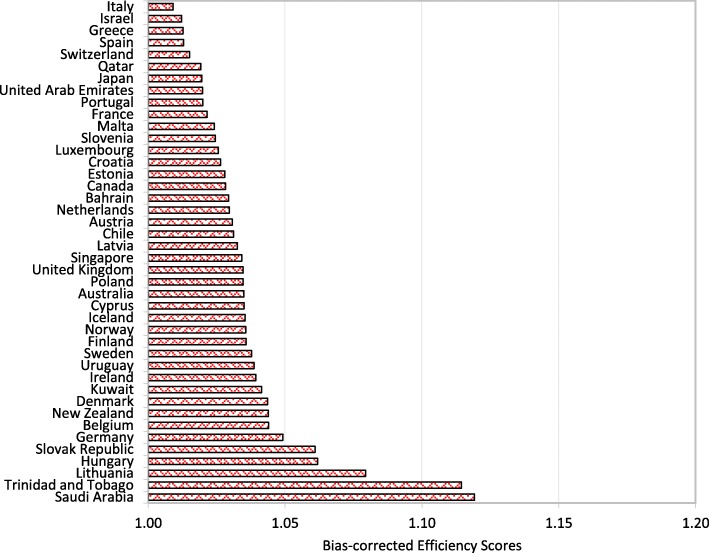
Global health system performance: High-income countries

**Fig. 2:**
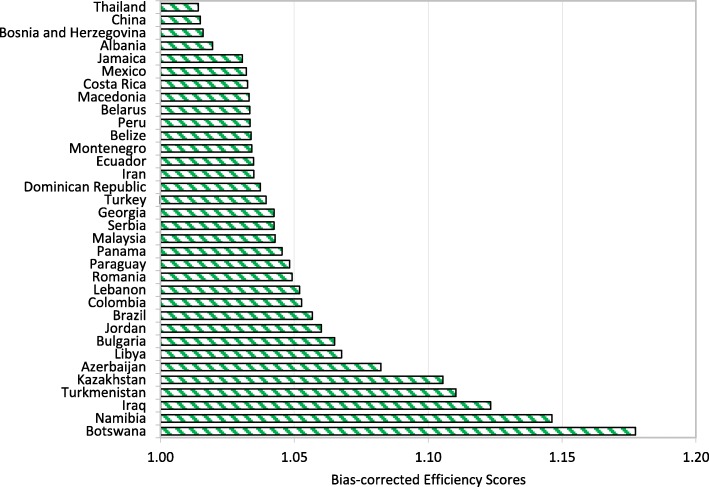
Global health system performance: Upper-middle-income countries

**Fig. 3:**
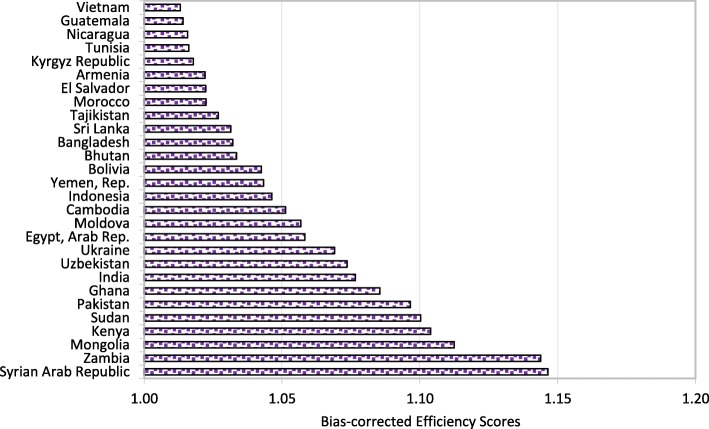
Global health system performance: Lower-middle-income countries

**Fig. 4:**
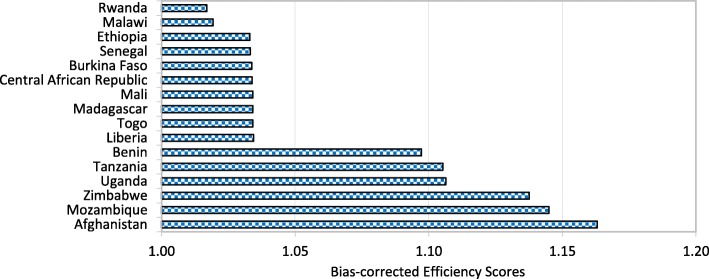
Global health system performance: Low-income countries

Figures 1, 2, 3 and 4 show that Italy, Israel, Greece (high-income countries), Thailand, China, Bosnia and Herzegovina (upper-middle-income countries), Vietnam, Guatemala, Nicaragua (lower-middle-income countries) Rwanda, Malawi and Ethiopia (low-income countries) had the most efficient health systems among their respective income groups in 2014. Therefore, the health systems of these countries are considered good references and offer useful information for the less efficient countries within the same income groups. For instance, among the developed countries, Canada has an efficiency score of 1.0283, which implies that the country’s health system should increase its outputs by at least 2.83% to reach full efficiency.

From Table A2 in Additional file 2, we can observe that Botswana, Cameroon, and Afghanistan obtained efficiency scores greater than 1.15 and were ranked at the bottom of the upper-middle-income, lower-middle-income and low-income groups, respectively. The results from the bootstrap DEA framework suggested that with existing country capacity and resources, more efficient utilisation of their resources in these countries could potentially enhance output by at least 15% in terms of both healthy life expectancy and an inverse mortality rate. This result arises partly because most underdeveloped and some developing countries are still lacking of healthcare accessibility and equity as well as service quality and efficiency. It is not surprising that the average efficiency scores for health systems in high-income and most upper-middle-income groups are better than the scores for lower-middle-income and low-income groups.

**Fig. 5:**
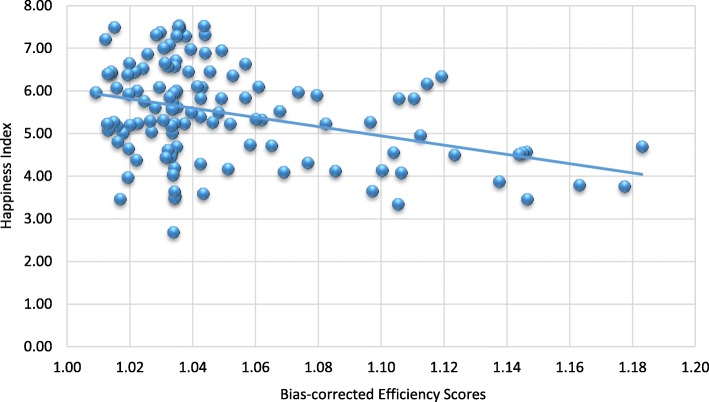
Happiness and health system performance

As stated in the previous section, the happiness index is a comprehensive indicator that includes several important components, such as, caring, freedom, generosity, honesty, health, income and good governance. The first happiness index was created in 2012 in the World Happiness Report. Many countries have started to promote happiness and well-being as a criterion for public policy. Does happiness matter to healthcare policy? An overall picture illustrating the relationship between the happiness index and health system efficiency is presented in Fig. 5. It is shown that happier societies tend to have better health system performance. However, a more robust analysis of the relationship between these two variables can be conducted using the second stage of the double bootstrap DEA framework.

### The results of second stage of double bootstrap DEA

In general, differences in the efficiency scores of selected countries’ health systems generated by DEA could be explained by some variables that were not included in the first-stage DEA. Location, lifestyle and socio-economic factors are common explanatory variables used in previous studies. In this study, happiness scores (HAPPINESS), population density (DENSITY), and healthcare share of GDP (GDP_SHARE) are included in the second stage of the double bootstrap DEA framework. Some other explanatory variables are excluded from the second stage of analysis partly due to collinearity problems (e.g., tobacco), data availability (e.g., readmission rate) and insignificant results (e.g., obesity).^3^ The results of the truncated regression are presented in Table 2. The dependent variable used in the truncated regression is a measure of health system inefficiency (i.e., ${\hat{\uptheta}}_{\mathrm{m}}^{\ast \ast }>1$). Hence, a positive coefficient indicates that an increase in a related explanatory variable leads to an increase in inefficiency of the country's health system.

**Table 2:** Results of double bootstrap DEA

Variables	Bootstrap coefficients	Bootstrap standard deviation	95% Bootstrap CI.^a^	
			Lower	Upper
HAPPINESS	−0.2478***	0.0315	−0.3123	−0.1905
DENSITY	−0.0187***	0.0048	−0.0287	−0.0096
GDP_SHARE	−0.0399**	0.0178	−0.0746	−0.0040

Notes

Dependent variable: Inefficiency scores (i.e. ${\hat{\uptheta}}_{\mathrm{m}}^{\ast \ast }>1$)

*C.I.* Confidence intervals

***and **represent statistical significance at levels 1 and 5% respectively

^a^ The figures are computed by 2000 bootstrap interactions

Happiness index (HAPPINESS) is included in the analysis to observe the contribution of happiness to the performance levels of selected global health systems. The relationship between happiness levels and health system inefficiency is found to be negative, which suggests that happier societies tend to have better health system performance. Being healthy can contribute to happiness, and improving happiness itself may also result in better health. Therefore, improving and sustaining the happiness of people is an alternative way to reduce a country’s healthcare expenditure and enhance health system performance. Many studies have proven that happiness is beneficial to health and can increase life expectancy.

Population density (DENSITY) is measured by the number of people per square kilometre of land area. The DENSITY variable is included to assess the effect of population density on health system performance. The coefficient of DENSITY is estimated and indicates a negative relationship between the population density and health system inefficiency level. This result is consistent with those obtained in earlier empirical studies, such as Greene [28] and Kumbhakar [30]. Therefore, countries with higher population density require more capital investment to increase healthcare outputs, including health protection.

The healthcare share of GDP (GDP_SHARE) is included in the second stage of the analysis. The estimated GDP_SHARE coefficient is negative, which indicates that countries with a higher healthcare share of GDP are likely to be more efficient than other countries. This difference occurs because healthcare costs have increased dramatically, with many countries experiencing increasing difficulties in sustaining their healthcare provision and performance. To obtain better health system outcomes, greater healthcare spending to GDP is essential, particularly, for those countries that fail to improve their level of happiness and lifestyle.

## Conclusions

This study employs the double bootstrap DEA approach to assess the health system efficiency of 121 selected countries based on the availability of data. It is not surprising that higher income and mostly developed countries have more efficient health systems. This study allows us to benchmark the health system efficiency of a group of countries according to their income levels. The results have useful implications for improving the health system efficiency of individual countries. Overall, the study shows that on average, the selected global health systems obtained an average bias-corrected technical score of 1.036 in 2014.

For the bootstrap truncated regression, similar studies have considered environment factors such as wealth, education level, and lifestyles (for example, [19, 30]) in the assessment of healthcare performance across nations. This study is one of the first to highlight the role of subjective well-being or happiness in healthcare performance. The findings show that happiness is one of the factors that contributes to the efficiency of a country’s health system. Many studies have also shown that happiness and health are inter-related, with happiness influencing health and health influencing happiness. Therefore, focusing policies on subjective well-being and happiness could lead to both improved well-being and improved health outcomes.

This study revealed that the selected health systems perform well, on average, in terms of characteristics such as population density and healthcare share of GDP. Health system capacity expansion can improve efficiency, but a larger scale of operations requires a large capital investment. Therefore, government and public support are relatively important for planning improvements to local health systems.

Economic growth on its own is no longer sufficient to increase the average level of happiness and well-being of people. Thus, in addition to improving the economic standard of living, policy-makers should also consider ways to increase happiness, social capital or income equality among the people. Looking at policy through the lens of well-being offers a fresh perspective for many nations worldwide. In healthcare, policies focusing on subjective well-being and happiness can lead to improved well-being and improved health outcomes, which may ultimately reduce the healthcare burden and enhance healthcare performance.

The selected variables and countries in this study are based on the data available from the WHO’s published reports and database. Some data are not available for certain variables in particular countries. Several data-related issues in this study need to be addressed in future research, and such issues include the different data measurements and methods used in the conversion of costs data. Furthermore, the current study offers a snapshot of only a single moment in time. More data points in different time periods are highly recommended in future research to observe efficiency improvements and productivity growth in global health systems.

The main objective of this study is to provide some empirical information related to the global health system performance. Apart from DEA model, SFA and price-based index number are alternative efficiency measurement tools which have been used in other efficiency studies. Each measurement tool has its own merits and limitations. Alternative models pertaining to efficiency studies are discussed by Coelli et al. [37]. Although DEA model is being adopted in this study, it would be useful to include sensitivity analysis involving SFA or price-based index number in future studies. However, we hope this study will initiate other attempts of similar direction into providing useful information for policy-makers in drafting health policies in the future.

## Additional files

Additional file 1: **Appendix A.** Previous health system efficiency studies. (PDF 537 kb)

Additional file 2: **Appendix B.** The results of global health system performance, 2014. (PDF 546 kb)

## Abbreviations

DEA: Data envelopment analysis
DMU: Decision making unit
GDP: Gross domestic product
OECD: Organisation for Economic Co-operation and Development
SFA: Stochastic frontier analysis
TE: Technical efficiency
VRS: Variable returns to scale
WHO: World Health Organisation

## Footnotes

1 Several common indicators such as income inequality, democratisation, freedom index and government effectiveness that were used by most researchers to explain the global health system performance (e.g. [28, 30]) are considered in constructing happiness index.

2 As for the case of nested data, multi-level regression could be a method that is worthy for future investigation. This regression model has yet to be applied in previous health system efficiency studies but it is definitely useful to be explored in future studies. We are grateful to a reviewer for this constructive comment.

3 Several diagnostic tests are used to examine multicollinearity across selected explanatory variables. The results of variance inflation factor and correlation analysis confirmed that there is no multicollinearity problem between happiness index, population density and healthcare share of gross domestic product.
