# Supplementary material for: Does happiness matter to health system efficiency? A performance analysis
Source: Health Econ Rev. 2018 Dec 21;8:33. doi: 10.1186/s13561-018-0214-6 (PMC6755568; doi:10.1186/s13561-018-0214-6)
Supplement: Supplementary file 1 — Appendix A. Previous health system efficiency studies. (PDF 537 kb) [file 13561_2018_214_MOESM1_ESM.pdf]

Appendix A: Previous health system efficiency studies.

Table A1. Summary of selected OECD and global health system efficiency studies

Author	Sample, Year	Method		Input Measure																			
		DEA	SFA	Age structure	Alcohol consumption	Discharges	Education	Fruits & vegetables consumption	Gross capital formation	Health employment	Health expenditure	Hospital beds	Income	Income inequality	Labour force	MRI	Non-physician personnel	Nurses	Pharmaceutical consumptions	Physicians	Physician consultations	Tobacco consumption	Pollution
OECD Health System Studies																							
de Cos and Moral-Benito [16]	29 OECD countries, 1997-2009		●		●			●			●		●									●	●
Hadad et al. [17]	31 OECD countries, 2007	●						●			●	●	●							●			
Wranik [18]	21 OECD countries, 1970-2008		●	●							●			●	●								
Afonso and Aubyn [19]	21 OECD countries, 2000-2003	●										●				●		●		●			
Spinks and Hollingsworth [20]	28 OECD countries, 1993-1997, 1995-2000	●					●				●		●										
Afonso and Aubyn [21]	24 OECD countries, 2000	●										●						●		●			
Bhat [22]	24 OECD countries, 1996	●										●						●	●	●			
Osterkamp [23]	29 OECD countries, 1980, 1990, 2000	●		●						●	●		●										
Retzlaff-Roberts et al. [24]	27 OECD countries, 1998	●					●				●	●		●		●				●		●	
Puig-Junoy [25]	All OECD countries except Turkey, 1961-1990	●		●	●							●					●			●		●	

Table A1 (cont...). Summary of selected OECD and global health system efficiency studies.

Author	Sample, Year	Method		Input Measure																			
		DEA	SFA	Age structure	Alcohol consumption	Discharges	Education	Fruits & vegetables consumption	Gross capital formation	Health employment	Health expenditure	Hospital beds	Income	Income inequality	Labour force	MRI	Non-physician personnel	Nurses	Pharmaceutical consumptions	Physicians	Physician consultations	Tobacco consumption	Pollution
Regional/Global Health System Studies																							
Sinimole [26]	180 WHO members, 2008	●									●							●		●			
Mirmirani and Lippmann [35]	13 countries of G12, 1991-1995	●					●				●	●				●				●			
Oglobin [27]	78 countries, 2000, 2003, 2007		●		●						●											●	
Greene [28]	191 countries, 1997		●				●				●												
Kumbhakar [30]	140 WHO members, 1993-1997		●				●				●												
Tajnikar and Dosennovic Bonca [36]	16 EU countries, 2000	●				●					●	●								●	●		
Grosskopf et al. [31]	143 countries, 1997	●					●		●		●				●								
Greene [29]	191 countries, 1993-1997		●				●				●												
Hollingsworth and Wildman [32]	191 countries, 1993-1997	●	●				●				●												

Table A1 (cont...). Summary of selected OECD and global health system efficiency studies.

Author	Sample, Year	Method		Output Measure									
		DEA	SFA	Age structure	Composite measure of health delivery	Disability-adjusted life expectancy	Discharges	Immunisation coverage	Income	Infant mortality	Life expectancy	Physician consultations	Potential years of life lost
OECD Health System Studies													
de Cos and Moral-Benito [16]	29 OECD countries, 1997-2009		●			●					●		
Hadad et al. [17]	31 OECD countries, 2007	●								●	●		
Wranik [18]	21 OECD countries, 1970-2008		●								●		
Afonso and Aubyn [19]	21 OECD countries, 2000-2003	●								●	●		●
Spinks and Hollingsworth [20]	28 OECD countries, 1993-1997, 1995-2000	●									●		
Afonso and Aubyn [21]	24 OECD countries, 2000	●								●	●		
Bhat [22]	24 OECD countries, 1996	●		●									
Osterkamp [23]	29 OECD countries, 1980, 1990, 2000	●											●
Retzlaff-Roberts et al. [24]	27 OECD countries, 1998	●								●	●		
Puig-Junoy [25]	All OECD countries except Turkey, 1961-1990	●									●		

Table A1 (cont...). Summary of selected OECD and global health system efficiency studies.

Author	Sample, Year	Method		Output Measure									
		DEA	SFA	Age structure	Composite measure of health delivery	Disability-adjusted life expectancy	Discharges	Immunisation coverage	Income	Infant mortality	Life expectancy	Physician consultations	Potential years of life lost
Regional/Global Health System Studies													
Sinimole [26]	180 WHO members, 2008	●						●		●			
Mirmirani and Lippmann [35]	13 countries of G12, 1991-1995	●								●	●		
Oglobin [27]	78 countries, 2000, 2003, 2007		●			●							
Greene [28]	191 countries, 1997		●		●								
Kumbhakar [30]	140 WHO members, 1993-1997		●			●							
Tajnikar and Dosennovic Bonca [36]	16 EU countries, 2000	●					●			●	●	●	
Grosskopf et al. [31]	143 countries, 1997	●							●	●	●		
Greene [29]	191 countries, 1993-1997		●		●	●							
Hollingsworth and Wildman [32]	191 countries, 1993-1997	●	●			●							

Notes: DEA: Data Envelopment Analysis; SFA: Stochastic Frontier Analysis
