# Supplementary material for: Does happiness matter to health system efficiency? A performance analysis
Source: Health Econ Rev. 2018 Dec 21;8:33. doi: 10.1186/s13561-018-0214-6 (PMC6755568; doi:10.1186/s13561-018-0214-6)
Supplement: Supplementary file 2 — Appendix B. The results of global health system performance, 2014. (PDF 546 kb) [file 13561_2018_214_MOESM2_ESM.pdf]

Appendix B: The results of global health system performance, 2014.

Table A2. Global health system performance: Original DEA scores, bias and bias-corrected efficiency scores

Country	Income Group	Original Score	Bias	Bias-corrected Score	Confidence Interval	
					Lower	Upper
Italy	High	1.0018	-0.0073	1.0091	1.0023	1.0235
Israel	High	1.0000	-0.0122	1.0122	1.0004	1.0278
Greece	High	1.0000	-0.0127	1.0127	1.0004	1.0274
Spain	High	1.0000	-0.0130	1.0130	1.0004	1.0367
Switzerland	High	1.0121	-0.0030	1.0151	1.0124	1.0219
Qatar	High	1.0040	-0.0152	1.0192	1.0045	1.0613
Japan	High	1.0000	-0.0196	1.0196	1.0005	1.0500
United Arab Emirates	High	1.0052	-0.0146	1.0198	1.0056	1.0491
Portugal	High	1.0000	-0.0200	1.0200	1.0004	1.0499
France	High	1.0168	-0.0047	1.0215	1.0173	1.0292
Malta	High	1.0000	-0.0241	1.0241	1.0004	1.0595
Slovenia	High	1.0000	-0.0245	1.0245	1.0004	1.0814
Luxembourg	High	1.0107	-0.0150	1.0256	1.0111	1.0810
Croatia	High	1.0199	-0.0066	1.0264	1.0203	1.0353
Estonia	High	1.0000	-0.0280	1.0280	1.0003	1.0954
Canada	High	1.0230	-0.0054	1.0283	1.0233	1.0445
Bahrain	High	1.0138	-0.0156	1.0294	1.0141	1.0619
Netherlands	High	1.0247	-0.0049	1.0297	1.0251	1.0379
Austria	High	1.0229	-0.0078	1.0307	1.0233	1.0426
Chile	High	1.0000	-0.0312	1.0312	1.0003	1.1096
Latvia	High	1.0209	-0.0117	1.0326	1.0213	1.0652
Singapore	High	1.0000	-0.0342	1.0342	1.0004	1.1396
United Kingdom	High	1.0283	-0.0063	1.0346	1.0288	1.0469
Poland	High	1.0299	-0.0048	1.0347	1.0302	1.0420
Australia	High	1.0301	-0.0048	1.0349	1.0304	1.0430
Cyprus	High	1.0000	-0.0351	1.0351	1.0004	1.1426
Iceland	High	1.0000	-0.0354	1.0354	1.0004	1.1377
Norway	High	1.0270	-0.0087	1.0357	1.0273	1.0576
Finland	High	1.0198	-0.0160	1.0358	1.0202	1.0863

Table A2 (cont...). Global health system performance: Original DEA scores, bias and bias-corrected efficiency scores.

Country	Income Group	Original Score	Bias	Bias-corrected Score	Confidence Interval	
					Lower	Upper
Sweden	High	1.0272	-0.0105	1.0377	1.0276	1.0684
Uruguay	High	1.0296	-0.0090	1.0387	1.0300	1.0534
Ireland	High	1.0307	-0.0086	1.0393	1.0311	1.0608
Kuwait	High	1.0314	-0.0101	1.0414	1.0317	1.0598
Denmark	High	1.0384	-0.0052	1.0436	1.0388	1.0580
New Zealand	High	1.0319	-0.0119	1.0438	1.0323	1.0743
Belgium	High	1.0371	-0.0069	1.0440	1.0375	1.0552
Germany	High	1.0439	-0.0053	1.0492	1.0443	1.0559
Slovak Republic	High	1.0550	-0.0060	1.0610	1.0554	1.0695
Hungary	High	1.0575	-0.0045	1.0620	1.0581	1.0689
Lithuania	High	1.0724	-0.0071	1.0795	1.0729	1.0920
Trinidad and Tobago	High	1.0999	-0.0145	1.1145	1.1003	1.1404
Saudi Arabia	High	1.1102	-0.0089	1.1192	1.1106	1.1324
Thailand	Upper-Middle	1.0054	-0.0088	1.0142	1.0058	1.0260
China	Upper-Middle	1.0000	-0.0149	1.0149	1.0003	1.0289
Bosnia & Herzegovina	Upper-Middle	1.0000	-0.0159	1.0159	1.0004	1.0363
Albania	Upper-Middle	1.0000	-0.0196	1.0196	1.0004	1.0568
Jamaica	Upper-Middle	1.0000	-0.0306	1.0306	1.0004	1.0927
Mexico	Upper-Middle	1.0244	-0.0077	1.0321	1.0247	1.0458
Costa Rica	Upper-Middle	1.0000	-0.0325	1.0325	1.0004	1.1131
Macedonia, FYR	Upper-Middle	1.0222	-0.0109	1.0331	1.0226	1.0527
Belarus	Upper-Middle	1.0000	-0.0334	1.0334	1.0005	1.1375
Peru	Upper-Middle	1.0243	-0.0092	1.0335	1.0247	1.0474
Belize	Upper-Middle	1.0000	-0.0339	1.0339	1.0004	1.1376
Montenegro	Upper-Middle	1.0000	-0.0341	1.0341	1.0004	1.1348
Ecuador	Upper-Middle	1.0264	-0.0084	1.0348	1.0268	1.0488
Iran	Upper-Middle	1.0000	-0.0349	1.0349	1.0006	1.1369
Dominican Republic	Upper-Middle	1.0268	-0.0105	1.0373	1.0272	1.0533

Table A2 (cont...). Global health system performance: Original DEA scores, bias and bias-corrected efficiency scores.

Country	Income Group	Original Score	Bias	Bias-corrected Score	Confidence Interval	
					Lower	Upper
Turkey	Upper-Middle	1.0351	-0.0044	1.0395	1.0355	1.0464
Georgia	Upper-Middle	1.0314	-0.0111	1.0424	1.0319	1.0629
Serbia	Upper-Middle	1.0372	-0.0052	1.0424	1.0376	1.0491
Malaysia	Upper-Middle	1.0332	-0.0097	1.0429	1.0336	1.0585
Panama	Upper-Middle	1.0347	-0.0108	1.0454	1.0351	1.0665
Paraguay	Upper-Middle	1.0349	-0.0133	1.0483	1.0353	1.0759
Romania	Upper-Middle	1.0445	-0.0047	1.0492	1.0448	1.0557
Lebanon	Upper-Middle	1.0434	-0.0086	1.0519	1.0437	1.0646
Colombia	Upper-Middle	1.0444	-0.0083	1.0528	1.0447	1.0678
Brazil	Upper-Middle	1.0507	-0.0061	1.0568	1.0510	1.0643
Jordan	Upper-Middle	1.0519	-0.0082	1.0601	1.0523	1.0735
Bulgaria	Upper-Middle	1.0605	-0.0046	1.0651	1.0608	1.0708
Libya	Upper-Middle	1.0592	-0.0085	1.0677	1.0596	1.0804
Azerbaijan	Upper-Middle	1.0773	-0.0050	1.0824	1.0777	1.0897
Kazakhstan	Upper-Middle	1.1018	-0.0037	1.1056	1.1022	1.1119
Turkmenistan	Upper-Middle	1.0996	-0.0108	1.1104	1.1000	1.1322
Iraq	Upper-Middle	1.1123	-0.0110	1.1233	1.1129	1.1417
Namibia	Upper-Middle	1.1310	-0.0152	1.1462	1.1315	1.1855
Botswana	Upper-Middle	1.1591	-0.0183	1.1775	1.1596	1.2126
Vietnam	Lower-Middle	1.0000	-0.0130	1.0130	1.0004	1.0300
Guatemala	Lower-Middle	1.0023	-0.0118	1.0141	1.0028	1.0344
Nicaragua	Lower-Middle	1.0000	-0.0158	1.0158	1.0004	1.0381
Tunisia	Lower-Middle	1.0081	-0.0080	1.0161	1.0084	1.0297
Kyrgyz Republic	Lower-Middle	1.0000	-0.0178	1.0178	1.0006	1.0410
Armenia	Lower-Middle	1.0000	-0.0221	1.0221	1.0004	1.0582
El Salvador	Lower-Middle	1.0000	-0.0223	1.0223	1.0003	1.0635
Morocco	Lower-Middle	1.0000	-0.0225	1.0225	1.0003	1.0525
Tajikistan	Lower-Middle	1.0143	-0.0126	1.0269	1.0147	1.0469

Table A2 (cont...). Global health system performance: Original DEA scores, bias and bias-corrected efficiency scores.

Country	Income Group	Original Score	Bias	Bias-corrected Score	Confidence Interval	
					Lower	Upper
Sri Lanka	Lower-Middle	1.0000	-0.0314	1.0314	1.0004	1.1119
Bangladesh	Lower-Middle	1.0000	-0.0321	1.0321	1.0005	1.1062
Bhutan	Lower-Middle	1.0000	-0.0335	1.0335	1.0004	1.1395
Bolivia	Lower-Middle	1.0309	-0.0116	1.0425	1.0315	1.0654
Yemen, Rep.	Lower-Middle	1.0294	-0.0139	1.0433	1.0299	1.0748
Indonesia	Lower-Middle	1.0345	-0.0119	1.0463	1.0350	1.0703
Cambodia	Lower-Middle	1.0379	-0.0134	1.0513	1.0383	1.0810
Moldova	Lower-Middle	1.0487	-0.0081	1.0568	1.0492	1.0740
Egypt, Arab Rep.	Lower-Middle	1.0431	-0.0152	1.0582	1.0435	1.0955
Ukraine	Lower-Middle	1.0605	-0.0085	1.0690	1.0609	1.0864
Uzbekistan	Lower-Middle	1.0663	-0.0072	1.0736	1.0667	1.0916
India	Lower-Middle	1.0675	-0.0092	1.0766	1.0679	1.1000
Ghana	Lower-Middle	1.0786	-0.0068	1.0854	1.0790	1.1025
Pakistan	Lower-Middle	1.0802	-0.0164	1.0965	1.0807	1.1451
Sudan	Lower-Middle	1.0893	-0.0109	1.1003	1.0897	1.1252
Kenya	Lower-Middle	1.0963	-0.0076	1.1039	1.0969	1.1147
Mongolia	Lower-Middle	1.0997	-0.0127	1.1124	1.1000	1.1378
Zambia	Lower-Middle	1.1345	-0.0093	1.1438	1.1350	1.1600
Syrian Arab Republic	Lower-Middle	1.1332	-0.0132	1.1464	1.1336	1.1676
Cameroon	Lower-Middle	1.1721	-0.0110	1.1830	1.1726	1.2059
Rwanda	Low	1.0011	-0.0159	1.0169	1.0015	1.0666
Malawi	Low	1.0034	-0.0159	1.0193	1.0038	1.0547
Ethiopia	Low	1.0000	-0.0331	1.0331	1.0003	1.1338
Senegal	Low	1.0000	-0.0332	1.0332	1.0004	1.1390
Burkina Faso	Low	1.0000	-0.0337	1.0337	1.0005	1.1393
Central African Republic	Low	1.0000	-0.0339	1.0339	1.0003	1.1379
Mali	Low	1.0000	-0.0342	1.0342	1.0005	1.1371
Madagascar	Low	1.0000	-0.0342	1.0342	1.0004	1.1355

Table A2 (cont...). Global health system performance: Original DEA scores, bias and bias-corrected efficiency scores.

Country	Income Group	Original Score	Bias	Bias-corrected Score	Confidence Interval	
					Lower	Upper
Togo	Low	1.0000	-0.0342	1.0342	1.0003	1.1432
Liberia	Low	1.0000	-0.0344	1.0344	1.0003	1.1416
Benin	Low	1.0791	-0.0181	1.0972	1.0796	1.1468
Tanzania	Low	1.0933	-0.0121	1.1053	1.0936	1.1372
Uganda	Low	1.0966	-0.0098	1.1064	1.0971	1.1326
Zimbabwe	Low	1.1246	-0.0130	1.1376	1.1250	1.1724
Mozambique	Low	1.1279	-0.0171	1.1450	1.1283	1.1779
Afghanistan	Low	1.1464	-0.0167	1.1631	1.1470	1.2081

Note: Income groupings of countries are based on the World Bank Income Classifications (2014-2015)
